# Characterization of English Braille Patterns Using Automated Tools and RICA Based Feature Extraction Methods

**DOI:** 10.3390/s22051836

**Published:** 2022-02-25

**Authors:** Sana Shokat, Rabia Riaz, Sanam Shahla Rizvi, Inayat Khan, Anand Paul

**Affiliations:** 1 Department of Computer Science and IT, University of Azad Jammu and Kashmir, Muzaffarabad 13100, Pakistan; snagul@yahoo.com (S.S.); rabiaiqbal18@gmail.com (R.R.); 2 Raptor Interactive (Pty) Ltd., Eco Boulevard, Witch Hazel Ave, Centurion 0157, South Africa; sanam_shahla@hotmail.com; 3 Department of Computer Science, University of Buner, Buner 19290, Pakistan; inayat_khan@uop.edu.pk; 4 The School of Computer Science and Engineering, Kyungpook National University, Daegu 41566, Korea

**Keywords:** machine learning, RICA features, PCA features, Braille patterns, visually impaired, SVM, KNN, Decision Tree, text conversion

## Abstract

Braille is used as a mode of communication all over the world. Technological advancements are transforming the way Braille is read and written. This study developed an English Braille pattern identification system using robust machine learning techniques using the English Braille Grade-1 dataset. English Braille Grade-1 dataset was collected using a touchscreen device from visually impaired students of the National Special Education School Muzaffarabad. For better visualization, the dataset was divided into two classes as class 1 (1–13) (a–m) and class 2 (14–26) (n–z) using 26 Braille English characters. A position-free braille text entry method was used to generate synthetic data. N = 2512 cases were included in the final dataset. Support Vector Machine (SVM), Decision Trees (DT) and K-Nearest Neighbor (KNN) with Reconstruction Independent Component Analysis (RICA) and PCA-based feature extraction methods were used for Braille to English character recognition. Compared to PCA, Random Forest (RF) algorithm and Sequential methods, better results were achieved using the RICA-based feature extraction method. The evaluation metrics used were the True Positive Rate (TPR), True Negative Rate (TNR), Positive Predictive Value (PPV), Negative Predictive Value (NPV), False Positive Rate (FPR), Total Accuracy, Area Under the Receiver Operating Curve (AUC) and F1-Score. A statistical test was also performed to justify the significance of the results.

## 1. Introduction

Visual impairment is defined as a loss of the ability to see that cannot be fixed with conventional procedures such as medication or glasses. In a report presented by the World Health Organization (WHO), 2.2 billion people all over the world suffer from near or distance vision problems [1]. Enormous efforts are required to assess the impact of illness on individuals and society. Recent advances in quantitative measures of the quality of life, life expectancy, the financial impact of disease and its treatment have allowed us to calculate the effects of illness and assist in future research to improve public health. Over 120 diseases and conditions have been thoroughly reviewed in terms of disability-adjusted life years (DALYs), quality-adjusted life years (QALYs), quality of life and financial measures [2]. Visual impairment harms the well-being of children and adults. School-aged children with vision impairments may have lower levels of academic achievement [3].

To educate visually impaired people, Louis Braille invented a language known as Braille, also known as night writing, which is a language introduced by Louis Braille specifically for visually impaired people [4]. Braille is composed of six dots. Using these different dots patterns, the visually impaired can read and write different characters. Previously, Braille was read and written using a slate and stylus. The world these days is equipped with the latest technologies that make people’s lifestyles more comfortable. Touchscreen devices are used more commonly; between 2009 and 2014, mobile screen readers’ popularity rose exponentially from 12% to 82% [5]. The use of touchscreen-based devices helped visually impaired people to live their lives in a better way. Touchscreens have shown remarkable developments in recent years. It encourages users to perform a wide range of tasks [6]. This includes e-learning, medical diagnosis, performing household chores, gaming, online shopping, etc. Touchscreens are easy to carry and simple to deal with, thus making them a leading tool used in our daily lives. People with visual impairments are an integral part of every community.

To live a better life, visually impaired people also adopt touchscreen devices for smoothly carrying out their daily activities [7]. Many famous applications are available for visually impaired users to help improve their living conditions, such as: “Look Tel”—the money identification mobile application, “Kurzweil-National Federation of the Blind Reader (KNFB) Reader” that reads any text aloud [8], “TapTapSee” that identifies objects using photos [9], “Color ID Free” that discovers the names of the colors around you [10] and “Be My Eyes”—the one that helps visually impaired people in real time [11]. Georgios et al. analyzed different assistive tools to highlight visually impaired people’s issues using these applications [12]. Braille, also known as night writing, is a language introduced by Louis Braille specifically for visually impaired people [13,14]. There are several touchscreen-based Braille applications like Braille Easy [14], Single Tap Braille [5], Braille Ecran [15], Braille Enter [16], Braille Sketch [17], etc. available for people with visual impairments. Visually impaired people face serious usability and accessibility issues with these applications. Research is progressing to develop practical and more friendly devices for visually impaired people. Recently, machine learning techniques have been widely used for converting Braille into natural language and vice versa. Currently, a lot of work is done for converting English into Braille [18,19,20,21,22,23,24,25]. However, these conversions are based on handwritten scanned Braille sheets. These procedures do not reduce the burden of visually impaired users.

**Figure 1:**
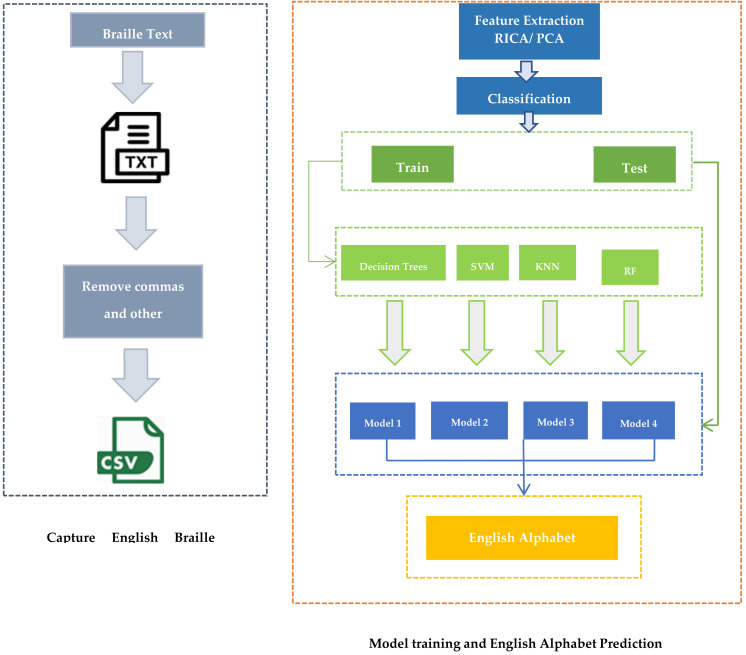
Schematic Diagram.

Braille to English character conversion has not been explored considerably; to the best of our knowledge, no current application for the visually impaired recognizes a position-free touchscreen-based Braille data entry in real time and provides both texts as well as voice feedback. Therefore, there is a strong need for an application that is both accessible to and usable for visually impaired users. The application proposed by the authors of Reference [19] is unique, because visually impaired users can tap anywhere on the screen and are not required to find the precise location of the dots. This work is the extension of a previous study conducted by Sana et al. The English Braille dataset used in this study was collected using the application proposed by Sana et al. In this research, the input storage mechanism was changed. Previously, Braille’s image was saved and used by the authors for character prediction. Here, the coordinate value of each dot entered is saved in a text file. The authors manually validated the numerical dataset acquired using the touchscreen application. Anomalies were removed before processing. Using this new dataset, Braille to English character recognition was made using machine learning techniques like DT, SVM and KNN combined with RICA- and PCA-based feature extraction methods. The Random Forest and Sequential methods were also implemented for a comparative analysis. Figure 1 represents the schematic diagram for the research conducted.

The following are the main contributions of this research paper:

(a) Collection of the English Braille Grade 1 dataset from visually impaired students of Muzaffarabad Special Education School, using the position-free Braille input application developed by Sana et al. This dataset was collected, as no such prior dataset exists that gathers Braille input from visually impaired users directly on touchscreen devices in real time.
(b) A novel backend storage mechanism for Braille characters entered using the touchscreen-based application.
(c) Prediction of the English alphabet for the corresponding Braille characters using Decision Tree (DT), Support Vector Machine (SVM), K-Nearest Neighbor (KNN) and Random Forest (RF) with RICA- and PCA-based feature extraction methods.
(d) Evaluation and comparison of the proposed mechanism with previous studies and using other techniques like the Random Forest and Sequential methods.

This manuscript is organized as follows: Section 2 provides a literature review, Section 3 presents the materials and methods, Section 4 contains the detailed results and comparative analysis, Section 5 contains the discussion and Section 6 contains the conclusions and future recommendations.

## 2. Literature Review

Braille is a language invented by Louis Braille, and it is still used worldwide as the standard communication tool for people with visual impairment. Braille is written by punching dots on paper and read by gliding the fingers over the raised dots. A Braille cell is defined by combining six dot patterns of 3 × 2 matrixes [26]. A couple of decades ago, efforts began to develop machines that could assist and speed up the writing process. Touchscreen devices, invented in 1965, have become an important part of everyday life. People can easily interact with touchscreens without using any other tools. Touchscreens enable developers to create interfaces tailored to the audience’s specific needs. A wide range of touchscreen-based applications are available to assist visually impaired people in the accomplishment of their daily life tasks with ease, like Braille Tap and Nav Tap [27], V-Braille [28], TypeIn Braille [29], Braille Touch [9,30], TapTapSee [9], Braille Play [31], Edge Braille [32], Braille Ecran [15], BeSpecular [33], Color Teller [34], KNFBReader [8], Text to Speech Synthesizer [35] and so on. Since Braille is a popular language for the visually impaired, converting Braille to other languages has become an important study area. Many researchers have used Braille in natural language conversion mechanisms by considering it an important research topic. Several techniques have been used in multiple studies to achieve better results for Braille in natural language predictions. Machine learning and Deep Learning techniques with different feature extraction methods were used in these studies. A study carried out by Hassan et al., a feed-forward neural network with 400 inputs in the input layer and 190 hidden neurons in the hidden layer, was designed to convert English books into Braille text. For this purpose, small and capital English alphabets, 10 numbers and space were used with and without noise removal. The neural networks achieved satisfactory results for converting text into Braille. The network was built with a simple structure that included several layers and neurons in each layer. Noisy input patterns were introduced to the network, including the noise of a standard 0.2 to all characters, resulting in one or two characters being detected incorrectly each time the program was run [18]. An automated value thresholding algorithm was used for accurately converting English text into Braille. After feeding English text to the system, every word was read and converted into Braille characters. With the advent of this method, visually impaired people can easily read novels, books, etc. Flexibility, low cost and portability were the major advantages of the system, but this system can only be used for reading purposes. Using this method, reading books becomes easier for visually impaired users [20]. Similarly, Braille character segmentation was performed using image processing techniques. Then, Support Vector Machine (SVM) with the Histogram of Oriented Gradients (HOG) feature extraction method was applied to translate English Braille characters. The Histogram of Oriented Gradients, or HOG, is a feature descriptor used to extract features from image data. It is generally used for image/object detection tasks. For this purpose, Megha et al. proposed another SVM technique to include a preprocessing step like noise removal and contrast enhancement on Braille images. Preprocessing was performed at each step to predict Braille to English characters accurately. A better performance achieved 96% accuracy using this technique. However, this study also worked only for scanned documents [23]. In a study by Raghunandan et al., an algorithm was designed that converts text to Braille and Braille to text. Raw data was captured in the form of images. After image acquisition and character segmentation, ASCII characters are converted to Braille text using newly designed algorithms. This method is also limited in the scanned-based input method [24]. The HAAR feature extraction method, along with SVM, was used by Li et al., using scanned Braille sheets for Braille to English conversion. The two-dimensional HAAR wavelet is often utilized in digital image processing as a feature extraction approach. Various versions of 2D HAAR wavelets detect edges in different orientations. Original, as well as cropped, images were used for training using the SVM. English characters were recognized successfully [36]. English characters were converted to the Braille system. Input characters were read using scanned files. Word segmentation was performed, and blank spaces were removed. The Braille database was accessed, the output was matched and the Braille characters were raised on a Braille pad [25]. English and Hindi text to Braille translation was performed. In a study carried out by Singh et al., English and Hindi words were read by the system. After removing spaces from the words and breaking them into the corresponding characters, they were matched using a lookup table [19]. A similar study was carried out to convert Scanned Braille English, Hindi and Tamil into text. For this purpose, 20 scanned Braille sheets were used. Ten were English Braille documents, five were Tamil Braille and five were Hindi Braille. Image enhancement and necessary noise removal were performed for converting Braille into text. A 100% accuracy was achieved by two English Braille documents and one Tamil Braille document. More than 97% accuracy was achieved for the rest of the documents [22]. An image-based Braille character recognition for English and Sinhala was performed using SVM with a Histogram of Oriented Gradient (HOG) feature extraction method. Characters were extracted after applying preprocessing steps and segmentation techniques. More than 99% accuracy was achieved for converting English- and Sinhala-based Braille characters [23]. The math to speech translation system made learning easy for people with visual disabilities. This tool helps non-native speakers and visually impaired people to solve mathematical equations easily [37,38,39].

Braille has been converted into other languages like Urdu [40,41], Arabic [42,43,44], Hindi [19,45], Bengali [46,47] Tamil [22], Sinhala [23], Kannada [48], ODIA [49], Chinese [50,51], Korean [52] and Gujarati [53,54,55].

In an earlier study conducted by the authors, a new touchscreen-based position-free application was developed that takes Braille input and converts it into equivalent Urdu characters. The English Braille dataset for this study was collected using the same application. This dataset was collected from the “National Special Education School”, Manak Payyan Muzaffarabad, Pakistan. Each student was requested to enter English Braille codes using the position-free application touchscreen device. This application was less tiring, because users had to tap only active dots for each character. This application can only recognize its equivalent English character based on the active dots entered. Every “X” and “Y” coordinate value of the tap dot is stored and further processed for character prediction.

In this research, the input storage mechanism was changed. Previously, Braille’s image was saved and used by the authors for character predictions. Here, the coordinate value of each dot entered was saved in a text file. The author manually validated the numerical dataset acquired using the touchscreen application. Anomalies were completely removed before processing. For Braille to English character recognition, machine learning techniques such as DT, SVM, KNN and RF are combined with the RICA- and PCA-based feature extraction methods. These techniques are simple to use to give better results, even with a small dataset.

## 3. Materials and Methods

### 3.1. Dataset Collection

**Figure 2:**
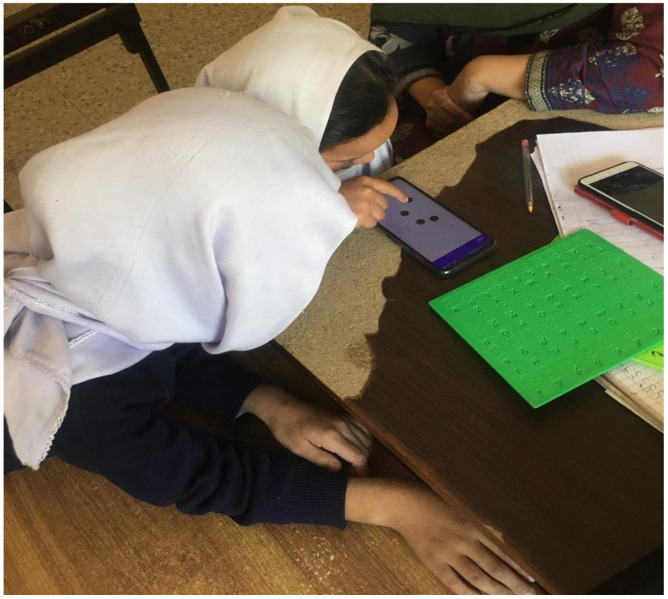
Visually Impaired student entering Braille patterns using a touchscreen device.

A position-free touchscreen-based braille input application was designed and developed by the authors in Reference [56]. Using this application, a new dataset of English Braille Patterns was collected from the students of the National Special Education School (NSEC) Manak Payyan, Muzaffarabad, Pakistan, as shown in Figure 2. The age of the students ranged between 12 and 19 years. Visually impaired students entered each English letter by tapping their fingers on the touchscreen. English Braille alphabets comprise different arrangements of six dots. Our application facilitates the users by requiring tapping of only the active dots for each character. “*x*”- and “*y*”-coordinate values of the tapped dots against one alphabet are stored with comma separators in a “.txt file”. The inactive dots are filled with a “0” value. All the comma separators are removed, and finally, after removing all the redundant data, a .csv file comprised of 2512 characters is made for further processing.

### 3.2. English Braille Character Recognition

This study used robust supervised machine learning techniques like SVM, KNN and Decision Trees to predict English Braille characters correctly. Even with small datasets, these algorithms are well-known for better text prediction [49]. These machine learning techniques were combined with the RICA-based feature extraction method, as it helps improve accuracy, reduce the risk of overfitting the model and speeds up the training process. The RICA (Reconstruction Independent Component Analysis method) also reduces the dimensionality by taking the input data as a mixture of independent components and correctly identifying each one by eliminating unnecessary noise.

The steps included for English Braille alphabet prediction are as follows:

Step 1: A “.csv” file is given as input for a machine learning application.

Step 2: Input data is divided into test and train data.

Step 3: 5-K cross-validation for (k = 5) was used.

Step 4: All three models are trained using training data.

Step 5: New models are generated for each applied algorithm.

Step 6: Performance of the newly built models are predicted using test data.

Step 7: English Braille alphabets are predated.

### 3.3. Feature Extraction

The first stage in classification is the extraction of features according to the type of problem. Previously, various feature extraction techniques used Braille to text predictions. Jha and Parvathi extracted the HOG feature for Braille to Hindi text conversion, and later, they were used by the SVM classifier [57]. In the same way, Li, Xeng and Zu used conventional extraction methods to identify Braille characters using KNN and the Naïve Bayes classifier [58]. Additionally, Li, Yan and Zhang employed HOG extractor methods using SVM to translate Braille to Odia, Sinhala and English [23,49]. The current study uses the reconstruction Independent Component Analysis (RICA)- and PCA-based feature extraction techniques with SVM, DT and KNN for Braille to English alphabet text translation.

#### 3.3.1. RICA Feature Extraction Method

RICA is not a supervised machine learning method; therefore, it does not require class label information. The RICA algorithm was implemented to address the shortcomings and disadvantages of the Independent Component Analysis (ICA) algorithm. This approach yielded more positive results than ICA. Many algorithms for learning sparse features have been introduced in the last few years. The sparse filter is being used to separate many natural signals and man-made ones, and this feature plays a significant role in various machine learning techniques.

The unlabeled data is indicated as the input.

(1) $${\left\{y^{\left(i\right)}\right\}}_{i=1}^{n},{\;y}^{\left(i\right)}\in \;{\mathbb{R}}^{m}$$

The problem of standard ICA [59] optimization for calculating independent components can be described mathematically as

(2)  X min1n∑i=1nhXyi

$$\mathrm{Subject}\;\mathrm{to}\;\ldots \;\ldots \;\ldots \;.{\mathrm{XX}}^{U}=I$$

where h(.) is a variation penalty function, “$X\in {\;S}^{L\;\times \;m}$” is a matrix-vector, L is the count of the vectors and “I” represents the identity matrix. Besides, ${\mathrm{XX}}^{U}=I$ is used to prevent the vectors in “X” from degenerating. A smooth penalty function can be used for this purpose, i.e., $h\left(.\right)=\log\left(\cos h\left(.\right)\right)$ [60]. On the other hand, the traditional Independent Component Analysis is blocked by some restrictions relevant to orthonormality from learning on an overcomplete basis. As a result, the deficiency mentioned above prevented ICA from scaling up to high-dimensional data. Therefore, soft reconstruction costs are used in RICA to cover orthonormality constraints in ICA. After this substitution, RICA filtering can be expressed with the following unconstrained problem:

(3)   X  minλn∑i=1n||XUXyi−yi ||22+∑i=1n∑k=1lhXkyi  

In the above equation, π > 0 indicates the tradeoff between sparsity and reconstruction error rate. RICA can learn sparse representations when X is over-completed after exchanging orthonormality constraints with reconstruction costs, even on unwhitened data in this way. However, penalty h can yield sparse, but not invariant, representations [61]. RICA [62] therefore swapped it with an additional pooling penalty defined by $L^{2}$, which, at the same time, helps to facilitate pooling features into associated features in the cluster. In addition, $L^{2}$ pooling also encourages sparsity for the learning of features. $L^{2}$ [63,64] pooling stands for a two-layered network in the first layer ${\left(.\right)}^{2}\;$ with square nonlinearity and nonlinearity in the second layer ${\left(\sqrt{\left(.\right)}\right)}^{2}$ square root.

(4) $$h\left({Xy}^{i}\right)=\overset{L}{\underset{k=1}{\sum }}\sqrt{{\varepsilon +H}_{k}\;.\left(\left({Xy}^{i}\right)⊙{(Xy}^{i})\right)}$$

In the pooling matrix $H\in P^{\left(L\times L\right)}$ by which $H_{k}$ reflects a row of the pooling matrix set to uniform weights, i.e., 1 for each component in the matrix H, element-wise multiplication is defined by a ⨀, and ε > 0 is a small average, as well as an element-wise multiplication. The RICA is a linear approach that only explores sparse representation in the real data space. The RICA method cannot use the relation between class label knowledge and sample training.

#### 3.3.2. Principal Component Analysis

PCA is one of the popular dimensionality reduction feature extraction methods. It is used for data exploration and predictive model development. It decreases the dataset’s dimensionality by merging several features into a few. While creating new features, PCA keeps the majority of the variance. PCA allows us to find correlations and patterns in a dataset to be converted into a dataset with relatively fewer dimensions while retaining all important data. PCA is the foundation of a projection-based multivariate data analysis. The most common application of PCA is to represent a multivariate data table as a smaller number of variables (summary indices) so that trends, jumps, clusters and outliers can be observed. PCA is a powerful technique that can evaluate datasets with missing values, categorical data and erroneous measurements. Its goal is to extract the most significant facts from the data and describe it as a set of summary indices known as principal components. The major advantage of using PCA on the dataset are removal of the correlation between the components, expediating the algorithm performance, resolving the problem of overfitting in the model and better visualization. However, due to feature reduction, there are chances of information loss [65].

### 3.4. Classification

Classification is the fundamental process for categorizing two or many classes according to the extracted features. Several machine learning methods are supervised, unsupervised, Ensemble Reinforced, Deep Learning and Neural Networks. Previously, most researchers used supervised Braille to text conversion learning techniques. Therefore, we used machine learning techniques such as Decision Trees, KNN and SVM based on the RICA extraction method [66].

#### 3.4.1. Decision Trees

Decision Trees are a predictive method used in machine learning. DT works well for both categorical and continuous data. Decision Trees require less effort to prepare data than other decision techniques. This is a standard classifier for machine learning, since it does not involve many computations [67]. Decision Tree classifiers have a tree-like structure, splitting the dataset into various subsets. This classifier trains the model by imposing basic rules on training data while making decisions [68]. Then, the model is used to predict and identify the targeted values by reading the dataset according to its classes [69].

Mathematically, a Decision Tree classifier can be formed using the following equations:

(5) $${\bar{X}=\{X}_{1}{,X}_{2}{,X}_{3},\ldots \ldots \ldots \ldots \ldots {.X}_{m}{\}}^{T}$$

(6) $$X_{i}=\left\{{\;x}_{1},x_{2},x_{3},\ldots \ldots .x_{\mathrm{ij}},\ldots \ldots \ldots x_{\mathrm{in}}\right\}$$

(7) $${\;S=\{S}_{1}{,S}_{2}{,\ldots \ldots \ldots S}_{i},\ldots \ldots ..S_{m}\}\;$$

In this analysis, we divided the test and training data with a ratio of 70:30. Using the training data is to construct a model, and test data is used to verify the model’s validity. This study used Decision Trees to predict Braille to English text using a multiclass approach. The default parameters were used to change the trees’ decisions.

#### 3.4.2. K-Nearest Neighbor

KNN is the most common and straightforward nonparametric technique employed in machine learning for regression and classification models. KNN is a simple algorithm that works well with smaller datasets, and only two parameters are required for implementation: the value of K and the distance function. The KNN algorithm does not require training before making predictions, and new data can be added without affecting the algorithm’s accuracy. The samples’ differences are determined using the following Euclidian Distance formula [67]:

(8) $${\mathrm{EU}}_{a,b}=\sqrt{\overset{n}{\underset{i=1}{\sum }}{\left(a_{i}-b_{i}\right)}^{2}}$$

where a and b represent the number of samples, and $\left(a_{i}-b_{i}\right)$ is the ith feature dimensions of the samples, and n denotes the total number of features dimension.

The Output value with the KNN classifier depends on the number of neighbors closest to it. If K = 1, the value means the object can be categorized and allocated to the nearest neighbor of that single class [68]. In this study, we used KNN to classify Braille into English text. We selected K = 3 in this analysis, with the Euclidean distance and equal weight.

#### 3.4.3. Random Forest

Random Forest is a supervised machine learning algorithm widely used in classification and regression problems. Random Forest is capable of handling both continuous and categorical data. It constructs Decision Trees from various samples and uses their majority vote for classification and average for regression, thus eliminating overfitting. It works well, even when the data contains null or missing values. Random Forest selects observations at random, builds a Decision Tree and uses the average result. It does not rely on any formulas. Different parameters are used for enhancing the predictive power and speed. Hyperparameters like n_estimators, max_features, mini-sample_leaf and n_jobs, random_state and oob_score are used to increase the predictive power and speed, respectively. Random Forest is more complex and requires more training time [70].

#### 3.4.4. Support Vector Machine

SVM is the most well-known machine learning classification algorithm for pattern identification and character recognition. SVM is a powerful classification technique for supervised data. It has been applied successfully to many applications, including Computer Vision, Biomedical Imaging and Speech Recognition, which require efficiently dealing with linear and nonlinear dimension data [71]. SVM is good at handling outliers and requires less training time and effort. To obtain better classification, SVM builds a hyperplane in high-dimensional areas. A classifier can achieve good efficiency if the hyperplane has a high functional margin [72]. A more significant margin reduces the risk of generalized mistakes. SVM finds the hyperplane, which provides the training data with the most significant minimum distance. SVM can produce a better, more generic performance. SVM is a double classifier that converts data into a hyperplane that depends on data of a higher dimension.

Let us consider hyperplane x. If w + b = 0, w is normal.

The representation of linearly separable data is as given below:

(9) $$\left\{x_{i},y_{i}\right\},{\;x}_{i\;}\epsilon R^{N}d,{\;y}_{i}\;\epsilon \left\{-1,1\right\},\;i=1,2,\ldots \ldots \ldots .N\;$$

where $y_{i}$ is the label of a two-fold class.

When we optimize the margin by maximizing the value of the objective function, E = ║w║^2^ gives

(10) $${\;x}_{i}.w+b\geq 1,{\;\mathrm{for}\;y}_{i}=+1$$

(11) $$\;\;x_{i}.w+b\;\leq 1,{\;\mathrm{for}\;y}_{i}=-1$$

By eliminating the inconsistencies of the above equations, we now have

(12) $$(x_{i}.b+b){\;y}_{i\;}\geq 1,\;\mathrm{for}\;\mathrm{all}\;i$$

If a dataset cannot be separated linearly, a slack variable “Ξi” is used to recognize classification errors. Thus, the objective function in this scenario is defined as

(13) $$\;E=\frac{1}{2}║w{║}^{2}+C\underset{i}{\sum }L\left(\Xi i\right)$$

Subject to

(14) $$(x_{i}.b+b)y_{i}\geq \;1-\xi_{i},\;\mathrm{for}\;\mathrm{all}\;i$$

Here, “C” and “L”. respectively, describe hyperparameters and cost functions. Cost functions for identifying outliers are used. The dual formulation with L(Ξi) = Ξi is

(15) $${\alpha =\max}_{\alpha }(\underset{i,j}{\sum }\alpha_{i}\alpha_{j}y_{i}y_{j}x_{i}x_{j})\;$$

Subject to

(16) $$0\leq \propto \alpha_{i}\leq C,\underset{i}{\sum }\alpha_{i}y_{j}=0\;\;\;$$

Here,

(17) $$\;\;\alpha =\left\{\alpha_{1},\alpha_{2},\alpha_{3}\ldots \ldots \ldots .\;\alpha_{i}\right\},\;\;\omega_{0\;\;}=\underset{i}{\sum }\alpha_{i}x_{i}y_{i}\;\;$$

A kernel trick is being used to accommodate separable data, which are not linear [73]. The nonlinear mapping function is transformed from the input space into a higher dimensional feature space. The dot product of $\left(x_{i},y_{i}\right)\;$ is replaced by functions to apply this kernel trick to two classes. The most popular kernels: Polynomial, Gaussian and Radial-based functions are given in Table 1.

The dual formation of a nonlinear case is shown as

(18) $$\alpha^{*\;}={\;\max}_{\alpha }(\underset{i}{\sum }\alpha_{i}+\underset{i,j}{\sum }\alpha_{i}\alpha_{j}y_{i}y_{j},K\left(x_{i}.y_{j}\right))$$

Subject to

(19) $$0\leq \alpha_{i}\leq C,\underset{i}{\sum }\alpha_{i}y_{j}=0$$

Grid search is the famous assessment metric used for evaluating SVM. The appropriate parameters are carefully selected by setting the grid range and phase size. Only one parameter, the “C” constant of soft margins, is used in a linear kernel, whereas SVM Gaussian kernel and SVM fine Gaussian kernel have two training parameters that cost “C” and Gamma to control the degree of nonlinearity. We used the RICA and PCA feature extraction methods with train test splits of 70:30 and 80:20, respectively. We applied default parameters to the Polynomial kernel.

#### 3.4.5. Sequential Model

The Sequential model is one of the simplest linear function neural network models. This model is suitable for a simple stack of layers with one input and one output tensor. In this model, not all the nodes are connected to other nodes of other layers. It handles input or output data sequences in text streams, audio clips, video clips, time series data and other types of sequential data. It comprises a convolution layer, nonlinear activation layer, pooling layer and a fully connected layer [74].

## 4. Results

English Braille alphabet recognition is performed using SVM, KNN and Decision Trees with the RICA-based feature extraction method. The performance metrics used for the evaluation are the True Positive Rate (TPR), True Negative Rate (TNR), False Positive Rate (FPR), Positive Predicted Value (PPV), Negative Predicted Value (NPV), Total Accuracy, Area Under the curve (AUC) and F1-Score.

**Figure 3:**
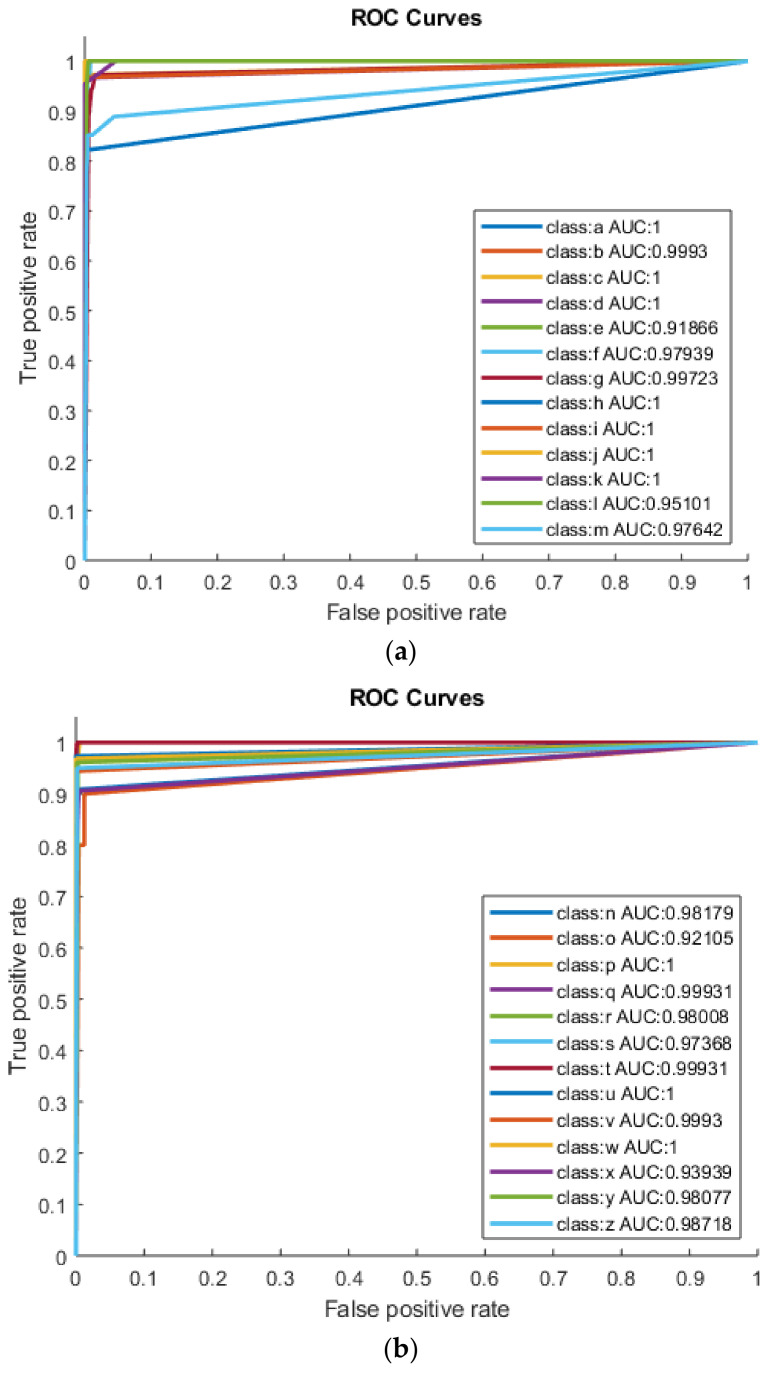
(**a**) Category-1 (a–m) using DT and (**b**) category-2 (n–z) using DT.

Figure 3a,b shows AUC values for category-1 (class a–class m) and category-2 (class n–class z) using DT. The highest performance achieved from category-1 (class a–class m) by using the RICA feature extraction method is for Braille classes a, c, d, h, i, j and k with TA (100%), TPR (100%), TNR (100%), AUC = 1 and F1-Score = 1. Following that, other classes like b and f achieved accuracy of 99.87% and 99.60%, AUC of 0.99 and 0.97 and F1-Score 0.99 and 0.94, respectively. As shown in Figure 3b, classes p, u and w from category-2 (n–z) have the best performances with TA (100%), TPR (100%), TNR (100%), AUC (1) and F1-Score = 1. Following them are classes q, s, t, v, y and z with TA (99.87%); TPR (100%, 94.74%, 100%, 96.15% and 97.44%); TNR (96.30%, 100%, 96.55%, 97.14%, 100% and 100%) and AUC (0.99, 0.97, 0.99, 0.99, 0.98 and 0.98). As shown in Table 2. the highest separation (AUC = 1) was achieved for the classes a, c, d, h, i, j, k, p, u and w. The AUC value of classes b, f, g, q, t and v is greater than 99.5%, indicating good classification.

**Figure 4:**
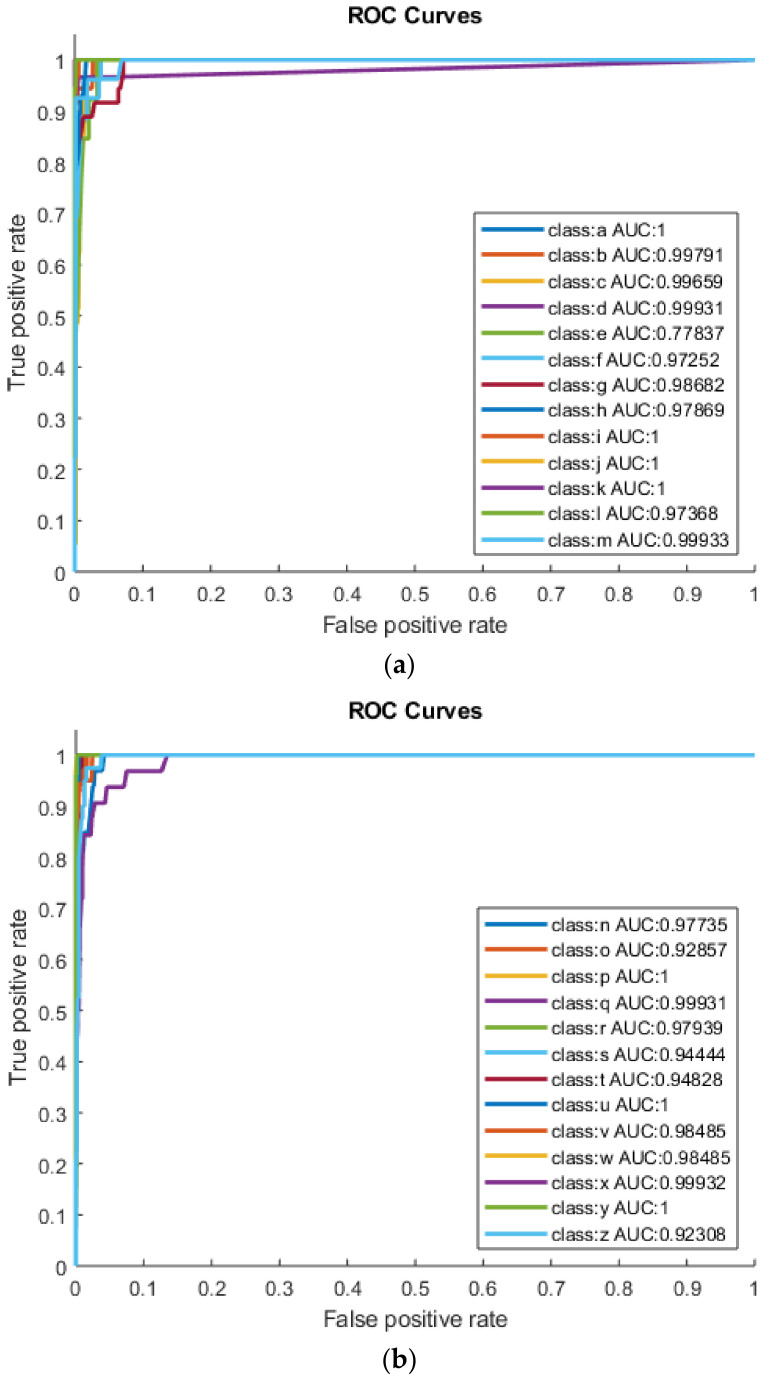
(**a**) Category-1 (a–m) using KNN and (**b**) category-2 (n–z) using KNN.

The highest accuracy achieved for category-1 (a-m) using KNN was for classes a, i, j and k, with TA (100%), TPR (100%), TNR (100%), AUC (1) and F1- Score achieved also equal to 1. As shown in Figure 4a, classes d, l and m have TA (99.87%); TPR (100%, 94.74% and 100%); TNR (99.86%, 100% and 99.87%) and AUC (0.99, 0.97 and 0.99), respectively. As shown in Figure 4b, the classes p, u and y achieve the highest TA of 100%, AUC and F1-Score equal to 1. For category-2 (n–z), followed by q, v, w and x with TA (99.87%); TPR (100%, 96.97%, 96.67% and 100%); TNR (99.86%, 100%, 100% and 99.86%); AUC (0.99, 0.98, 0.98 and 0.99) and F1-Score (0.98, 0.98, 0.98 and 0.97). The overall results indicated that English Braille characters such as a, i, j, k, p, u and y achieved the highest AUC value of 1, indicating 100% separation. Using KNN on the extracted feature set yielded AUC values greater than 0.99 for English Braille characters such as b, c, d, m, q and x. KNN also showed promising results for recognizing Urdu Braille characters. It has also improved the results for English Braille alphabet recognition. Table 3 exhibits detailed KNN results for all the characters.

**Figure 5:**
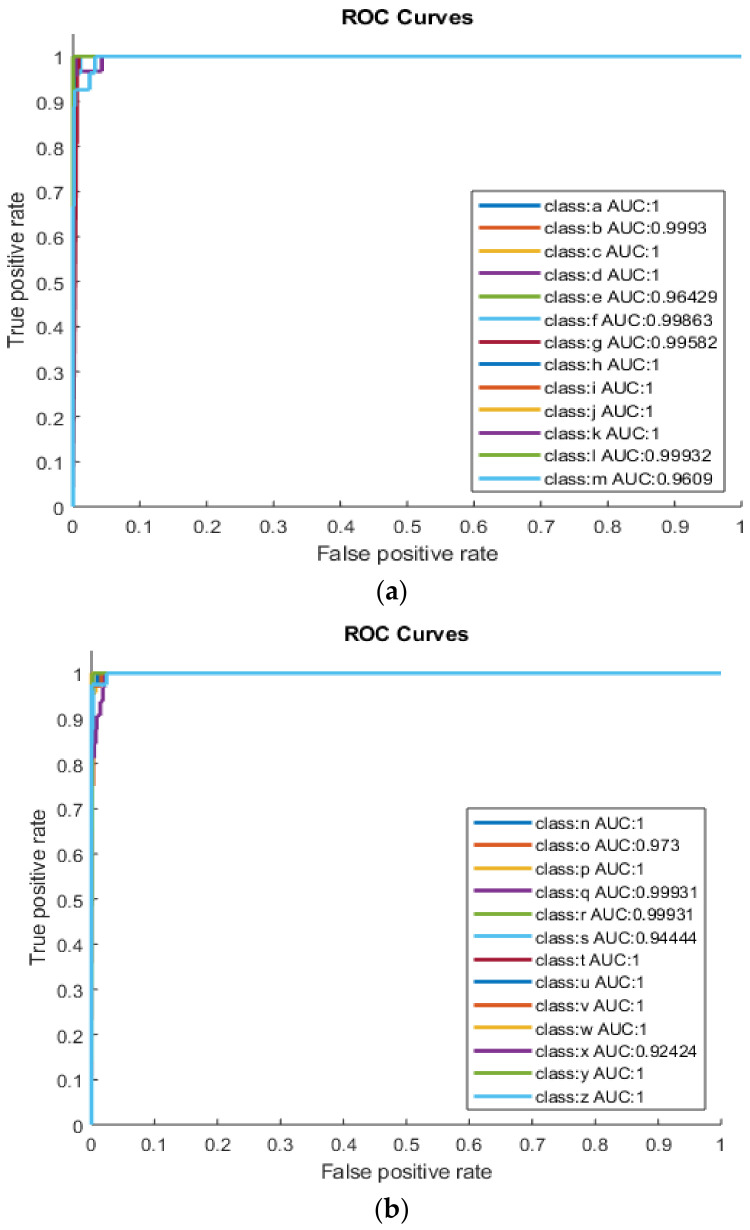
(**a**)Category-1 (a–m) using SVM and (**b**) category-2 (n–z) using SVM.

Furthermore, SVM was used to evaluate performances of different classes. Category-1 (a–m) achieved the highest accuracy with TA, TPR and TNR (100%) and the highest separation AUC of 1 and F1-Score also 1 for classes a, c, d, h, i, j and k. Followed by b and l with TA (99.87%), TPR (100%), TNR (99.86%) and AUC (0.99), as shown in Figure 5a. The highest performance for category-2 (n–z) was achieved for classes n, p, t, u, v, w, y and z with the TA, TPR and TNR (100%) and AUC (1) and F1-Score also 1. As shown in Figure 5b, classes q and r with TA (99.87%), TPR (100%), TNR (99.86%) and AUC (0.99) are followed by classes o and s with TA (99.73%), TPR (94.74% and 88.89%), TNR (99.86% and 100%), AUC (0.97 and 0.94) and F1-Score (0.95 and 0.94), respectively. Among all classes, a, c, d, h, i, j, k, n, p, t, u, v, w, y and z have the greatest separation of 1. Table 4 presents the SVM classifier’s detailed performance for English Braille character recognition.

For comparison purposes, we used PCA-based feature extraction methods, Random Forest and the Simple neural network. Among all the results, classifiers used with the RICA-based feature extraction method showed the best performances. Using Random Forest with five n_folds, a max_depth of 10 and Trees value of 10, the mean accuracy achieved was 75.61%. The results revealed by implementing the Sequential model using activation functions ReLu and Softmax, a batch size of five and 2000 epochs accuracy achieved was 93.51%, with a loss of 0.1736. With PCA feature extraction, the accuracy achieved for SVM, KNN and DT were 86.32%, 75.40% and 70.02%, respectively. All performances of SVM were better than DT and KNN using both feature extraction methods. SVM achieved a TA of (99.86%), TPR of (98.23%), TNR of (99.91%), PPV of (97.94%), NPV of (99.94%), FPR of (0.0009%) and F1-Score = 0.980. Followed by DT, which shows the TA (99.79%), TPR (96.91%), TNR (99.89%), PPV (97.22%), NPV (99.89%), FPR (0.0011) and F1-score (0.970), followed by KNN with TA (99.50%), TPR (95.32%), TNR (99.70%), PPV (93.70%), NPV (99.79%), FPR (0.0030%) and F1-Score (0.939), as shown in Table 5.

Two hypotheses were built to measure the significance between the results achieved using RICA- and PCA-based feature extraction method on different classification techniques. A *t*-test was applied to calculate the *p*-value.

**Hypothesis** **1** **(Null** **Hypothesis).** *There is no difference in the results of the RICA-based features extraction method using DT, KNN, SVM and RF.*

**Hypothesis** **1** **(Alternative** **Hypothesis).** *There is a difference between the results of the RICA-based feature extraction method using DT, KNN, SVM and RF.*

**Hypothesis** **2** **(Null** **Hypothesis).** *There is no difference in the results of the PCA-based features extraction method using DT, KNN, SVM and RF.*

**Hypothesis** **2** **(Alternative** **Hypothesis).** *There is a difference between the results of the PCA-based feature extraction method using DT, KNN, SVM and RF.*

The results of the RICA-based feature extraction method showed that there is a significant difference among the values of DT vs. KNN and DT vs. RF, as the *p*-value was less than 0.05, but no significant difference was found between SVM and DT. For the PCA-based feature extraction method, a significant difference was observed in all three comparisons. The results are shown in Table 6.

## 5. Discussion

Braille to natural language conversion is essential for people with visual impairment. This can help to improve their living quality. English is the most common medium used for communication all over the world. Various studies have been carried out for converting Braille into English. In the literature, several studies have converted Braille to English or vice versa. However, those studies usually used the conventional methods for text conversion. Previous studies used handwritten scanned Braille sheets a thes input, and then, those scanned sheets were converted into other languages. Padmavathi et al. used English, Hindi and Tamil handwritten scanned sheets for Braille conversion [22].

Similarly, Perera et al. used scanned Braille sheets using a histogram of gradient features and SVM [23]. Most of the studies used conventional methods like scanned-based input with different character prediction techniques like Deep Learning; Image classification and other machine learning techniques like SVM, KNN, Artificial Neural Network (ANN), etc. These classification techniques were used with different feature extraction methods for character recognition, as shown in Table 7. Whereas we used touchscreen-based real-time Braille input for Braille to natural language conversion, the focus of this study was to provide users with a position-free Braille input method using touchscreen-based android devices to provide an improved Braille to English Language conversion mechanism that would be more user-friendly and easily accessible.

For the prediction of Braille to English characters, DT, SVM and KNN, along with RICA- and PCA-based feature extraction, were used. The English Braille dataset was collected from visually impaired students at the National Special Education School using a previously developed position-free touchscreen-based application. SVM, KNN and DT showed better performances using the RICA feature extraction method. The major findings were achieved using DT for the highest TA for characters: a, c, d, h, i, j, k, p, u and w, yielding TA = 100%, AUC = 1 and F1-Score = 1. Characters like b, m, q, s, t, v, y and z were next, with TA = 99.87%; AUC of 0.999, 0.976, 0.999, 0.974, 0.999, 0.999, 0.981 and 0.985 and F1-Scores 0.99, 0.98, 0.97, 0.98, 0.99, 0.98 and 0.99, respectively. The maximum accuracy was achieved with the KNN classifier for characters a, i, j, k, p, u and y, with TA, TPR and TNR = 100%, AUC = 1 and F1-Score = 1. This was followed by characters d, l, m, q, v, w and x with TA = 99.87%; TPR = 100%, 99.74%, 100%, 100%, 96.97%, 96.97%, and 100%; TNR > 99%; AUC 0.999, 0.974, 0.999, 0.985, 0.985 and 0.999 and F1-Score 0.98, 0.97, 0.96, 0.98, 0.98, 0.98 and 0.97, respectively. Similarly, the SVM classifier achieved the best performance for classes a, c, d, h, i, j, k, n, p, t, u, v, w, y and z with TA, TPR, TNR = 100% and with F1-Score = 1, and the highest separation of 1 was achieved. Classes b, l, q and r were next, with TA = 99.87%; TPR = 100%; TNR = 99.86%, 99.86%, 100% and 99.96%; AUC 0.999, 0.999, 1 and 0.999 and F1-Scores 0.99, 0.97, 0.98 and 0.98, respectively. The total accuracy achieved using PCA with SVM, KNN and DT were 86.32%, 75.40% and 70.02%, respectively. Other results achieved were Precision (88.12%, 79.56% and 72.01%); Recall (86.32%, 75.45% and 68.04%) and F1- Score (0.86, 0.75 and 0.71). For comparison purposes, Random Forest with the RICA and PCA and Sequential models has also been employed, and they achieved accuracies of 80%, 90.02% and 93.51%, respectively.

**Table 7:** Comparative analysis with previous studies.

Paper Title	Input Method	Supported Language	Braille to Text	Text to Braille	Techniques Used	Feature Extraction/Algorithms/Others	Accuracy	Reference
Braille Messenger: Adaptive Learning Based Non-Visual Touchscreen Text Input for the Blind Community Using Braille	Gesture-Based Touchscreen Input	English	Yes	No	KNN	Bayesian Touch Distance	97.4%	[73]
					Nill	Newly Proposed Static Mathematical Algorithm	94.86%	
Conversion of Braille to Text in English, Hindi, And Tamil Languages	Hand-Written Scanned Braille Sheets	English	Yes	No	Nill	Image Segmentation Technique	99.4%	[22]
Optical Braille Recognition with HAAR Wavelet Features and Support-Vector Machine	Hand-Written Scanned Braille Sheets	English	Yes	No	SVM	HAAR Feature Extraction Method	Reduced classification error to 10	[36]
Optical Braille Recognition Based on Histogram of Oriented Gradient Features and Support-Vector Machine	Hand-Written Scanned Braille Sheet	English	Yes	No	SVM	HOG Feature Extraction Method	99%	[23]
Robust Braille recognition system using image preprocessing and feature extraction algorithms	Hand-Written Braille Scanned Sheet	English	Yes	No	Image Processing Techniques	Edge Detection, Image Projection, and Image Segmentation	100%	[75]
Braille Identification System Using Artificial Neural Networks	Hand-Written Braille Scanned Sheet	English	Yes	No	Artificial Neural Network	Back Propagation Algorithm	85%	[76]
Conversion Of English Characters Into Braille Using Neural Network	Hand-Written English Scanned Sheet	English	No	Yes	Neural Network		Noise with 0.1 std showed no errors	[18]
Designing Of English Text To Braille Conversion System: A Survey	Hand-Written English Scanned Sheet	English	No	Yes	Microcontroller		Accurate mapping of English to Braille text	[20]
Efficient Approach for English Braille to Text Conversion	Hand-Written English Scanned Sheet	English	Yes	No	SVM	Image Enhancement, Noise Reduction, Contrast Enhancement and Image Dilation	96%	[21]
The Methods Used in Text to Braille Conversion and Vice Versa	Image Taken from Camera	English	No	Yes	Raspberry PI		Accurate output was achieved	[24]
Automated Conversion of English and Hindi Text to Braille Representation	Hand-Written Scanned Sheets	English	No	Yes		Using Lookup Tables	English To Braille characters were accurately mapped	[19]
Application of Deep Learning to Classification of Braille Dot for Restoration of Old Braille Books	Hand-Written Braille Scanned Sheets	Braille			Deep Learning	Image Enhancement and Restoration Techniques	98%	[77]
A Recurrent Neural Network Approach to Image Captioning in Braille for Blind-Deaf People	English Captions of Images Taken from Camera	English			Deep Recurrent Neural Network		BLEU-4 Score Of 0.24 is achieved	[78]
Smart Braille Recognition System	Braille Images Taken from Camera	English	Yes	No	Bayesian	Centroid Features	100%	[79]
					KNN		100%	
					Classification Tree		80.76%	
					SVM		67.9%	
Proposed Schemes	Touch-Screen Based Input Method	English	Yes	No	SVM	RICA Feature Extraction	99.86%	
					KNN		99.50%	
					DT		99.79%	
					RF		90.02%	
					SVM	PCA Feature Extraction	86%	
					KNN		75%	
					DT		70.02%	
					RF		80%	
					Sequential Method		93.51%	

## 6. Conclusions and Future Work

Braille is used as an important means of communication for people with low or no vision. There are approximately over 150 million Braille users worldwide. Braille is becoming increasingly accessible to blind or visually challenged persons with the growing use of technology. Numerous studies have been carried out for Braille to English conversion. The majority of these conversions are carried out with scanned sheets as the input. This research collected a new English Braille dataset using touchscreen devices. The authors used the Android application developed to collect Braille English datasets in their previous research. For visually impaired users, the application is less tiring and less complicated. Machine learning techniques such as SVM with polynomial kernels, KNN = 3 and Decision Trees with default parameters are combined with RICA- and PCA-based feature extraction methods for English alphabet recognition. For training and testing, the dataset was split into 70:30 and 80:20, respectively. Precision, Recall, F1-Score and Accuracy were used as the evaluation metrics using PCA SVM, KNN and DT accuracies of 86.32%, 75.40% and 70.02%, whereas better results were obtained using RICA with SVM, KNN and DT. The SVM classifier outperformed all others, achieving an accuracy of 99.85%. KNN and DT achieved 99.50% and 99.79% accuracy, respectively. For comparing these results with other techniques, Random Forest with the RICA and PCA and Sequential methods were used, and they achieved an accuracy of 90.01% and 80%, respectively.

This work was limited to only Grade 1 Braille for the English language. This work can be enhanced by increasing the number of datasets not only for Grade 1 but also for Grade 2 and contracted Braille. Currently, the results of the proposed models are not satisfactory when implemented on mobile devices with limited computation power; thus, we tested these results only for computers. The future plan includes converting these models into lighter versions to work appropriately on Android devices. This study used RICA- and PCA-based feature extraction methods for English Braille character prediction with robust machine learning techniques on the Grade 1 Braille dataset. This work can be enhanced to the Braille English dataset of Grade 2. Deep learning techniques like CNN, GoogLeNet and Transfer Learning will improve the results for mobile devices.

## Figures and Tables

**Table 1:** SVM kernel descriptions.

Kernel Type	Classification Method	Mathematical Description
Linear Kernel	Linear SVM	$K\left(x_{i},y_{i}\right)\;=\;\left(x_{i},y_{i}\right)$
Polynomial Kernel	Quadratic SVM	$K\left(x_{i},y_{i}\right)\;={\left(1+x_{i},y_{i}\right)}^{2}$
	Cubic SVM	$K\left(x_{i},y_{i}\right)\;={\left(1+x_{i},y_{i}\right)}^{3}$
Gaussian Radial Base Function	Fine Gaussian SVM	$K\left(x_{i},y_{i}\right)\;=\;\exp\left(\frac{\left||x_{i}-y_{i}{}^{2}|\right|}{2\sigma^{2}}\right),\;\sigma =0.75$
	Medium Gaussian SVM	$K\left(x_{i},y_{i}\right)\exp\left(\frac{\left||x_{i}-y_{i}{}^{2}|\right|}{2\sigma^{2}}\right),\;\sigma =3$
	Course Gaussian SVM	$K\left(x_{i},y_{i}\right)\;=\exp\left(\frac{\left||x_{i}-y_{i}{}^{2}|\right|}{2\sigma^{2}}\right),\;\sigma =12$

**Table 2:** Performance metrics showing the results obtained for the Decision Tree classifier.

Serial Number	English Characters	TPR (%)	TNR (%)	PPV (%)	NPV (%)	FPR (%)	Total Accuracy (%)	AUC	F1-Score
1	a	100	100	100	100	0.00	100	1.00	1.00
2	b	100	99.86	97.14	100	0.14	99.87	0.99	0.99
3	c	100	100	100	100	0.00	100	1.00	1.00
4	d	100	100	100	100	0.00	100	1.00	1.00
5	e	83.87	99.86	96.30	99.31	0.14	99.20	0.91	0.90
6	f	96.15	99.72	92.59	99.86	0.28	99.60	0.97	0.94
7	g	100	99.45	88.89	100	0.55	99.47	0.99	0.94
8	h	100	100	100	100	0.00	100	1.00	1.00
9	i	100	100	100	100	0.00	100	1.00	1.00
10	j	100	100	100	100	0.00	100	1.00	1.00
11	k	100	100	100	100	0.00	100	1.00	1.00
12	l	90.48	99.73	90.48	99.73	0.27	99.47	0.95	0.90
13	m	95.83	99.45	85.19	99.86	0.55	99.34	0.97	0.90
14	n	96.77	99.58	90.91	99.86	0.42	99.47	0.98	0.94
15	o	84.21	100	100	99.59	0.00	99.60	0.92	0.91
16	p	100	100	100	100	0.00	100	1.00	1.00
17	q	100	99.86	96.30	100	0.14	99.87	0.99	0.98
18	r	96.15	99.86	96.15	99.86	0.14	99.73	0.98	0.96
19	s	94.74	100	100	99.86	0.00	99.87	0.97	0.97
20	t	100	99.86	96.55	100	0.14	99.87	0.99	0.98
21	u	100	100	100	100	0.00	100	1.00	1.00
22	v	100	99.86	97.14	100	0.14	99.87	0.99	0.99
23	w	100	100	100	100	0.00	100	1.00	1.00
24	x	87.88	100	100	99.45	0.00	99.47	0.93	0.94
25	y	96.15	100	100	99.86	0.00	99.87	0.98	0.98
26	z	97.44	100	100	99.86	0.00	99.87	0.98	0.99
26	z	97.44	100	100	99.86	0.00	99.87	0.98	0.99

**Table 3:** Performance metrics showing the results obtained for the KNN classifier.

Serial Number	English Characters	TPR (%)	TNR (%)	PPV (%)	NPV (%)	FPR (%)	Total Accuracy (%)	AUC	F1-Score
1	a	100	100	100	100	0.00	100	1.00	1.00
2	b	100	99.58	91.89	100	0.42	99.60	0.99	0.96
3	c	100	99.32	80.00	100	0.68	99.34	0.99	0.89
4	d	100	99.86	96.67	100	0.14	99.87	0.99	0.98
5	e	55.81	99.86	96.00	97.39	0.14	97.34	0.77	0.71
6	f	96.15	98.35	67.57	99.86	1.65	98.27	0.97	0.79
7	g	100	97.36	62.75	100	2.64	97.48	0.98	0.77
8	h	96.43	99.31	84.38	99.86	0.69	99.20	0.98	0.90
9	i	100	100	100	100	0.00	100	1.00	1.00
10	j	100	100	100	100	0.00	100	1.00	1.00
11	k	100	100	100	100	0.00	100	1.00	1.00
12	l	94.74	100	100	99.86	0.00	99.87	0.97	0.97
13	m	100	99.87	92.31	100	0.13	99.87	0.99	0.96
14	n	96.30	99.17	81.25	99.86	0.83	99.07	0.97	0.88
15	o	85.71	100	100	99.59	0.00	99.60	0.93	0.92
16	p	100	100	100	100	0.00	100	1.00	1.00
17	q	100	99.86	96.43	100	0.14	99.87	0.99	0.98
18	r	96.15	99.72	92.59	99.86	0.28	99.60	0.98	0.94
19	s	88.89	100	100	99.73	0.00	99.73	0.94	0.94
20	t	89.66	100	100	99.59	0.00	99.60	0.95	0.95
21	u	100	100	100	100	0.00	100	1.00	1.00
22	v	96.97	100	100	99.86	0.00	99.87	0.98	0.98
23	w	96.97	100	100	99.86	0.00	99.87	0.98	0.98
24	x	100	99.86	94.44	100	0.14	99.87	0.99	0.97
25	y	100	100	100	100	0.00	100	1.00	1.00
26	z	84.62	100	100	99.17	0.00	99.20	0.92	0.92

**Table 4:** Performance metrics showing the results obtained for the SVM classifier.

Serial Number	English Characters	TPR (%)	TNR (%)	PPV (%)	NPV (%)	FPR (%)	Total Accuracy (%)	AUC	F1-Score
1	a	100	100	100	100	0.00	100	1.00	1.00
2	b	100	99.86	97.30	100	0.14	99.87	0.99	0.99
3	c	100	100	100	100	0.00	100	1.00	1.00
4	d	100	100	100	100	0.00	100	1.00	1.00
5	e	92.86	100	100	99.72	0.00	99.73	0.96	0.96
6	f	100	99.73	92.00	100	0.27	99.73	0.99	0.96
7	g	100	99.16	85.37	100	0.84	99.20	0.99	0.92
8	h	100	100	100	100	0.00	100	1.00	1.00
9	i	100	100	100	100	0.00	100	1.00	1.00
10	j	100	100	100	100	0.00	100	1.00	1.00
11	k	100	100	100	100	0.00	100	1.00	1.00
12	l	100	99.86	95.00	100	0.14	99.87	0.99	0.97
13	m	92.59	99.59	89.29	99.72	0.41	99.34	0.96	0.91
14	n	100	100	100	100	0.00	100	1.00	1.00
15	o	94.74	99.86	94.74	99.86	0.14	99.73	0.97	0.95
16	p	100	100	100	100	0.00	100	1.00	1.00
17	q	100	99.86	96.43	100	0.14	99.87	0.99	0.98
18	r	100	99.86	96.30	100	0.14	99.87	0.99	0.98
19	s	88.89	100	100	99.73	0.00	99.73	0.94	0.94
20	t	100	100	100	100	0.00	100	1.00	1.00
21	u	100	100	100	100	0.00	100	1.00	1.00
22	v	100	100	100	100	0.00	100	1.00	1.00
23	w	100	100	100	100	0.00	100	1.00	1.00
24	x	84.85	100	100	99.31	0.00	99.34	0.92	0.92
25	y	100	100	100	100	0.00	100	1.00	1.00
26	z	100	100	100	100	0.00	100	1.00	1.00

**Table 5:** Overall results achieved using Decision Trees, SVM and KNN with the RICA and PCA feature extraction methods.

Classifier	Feature Extraction Method	Precision (%)	Recall (%)	F1-Score	Accuracy (%)
DT	RICA	97.22	96.91	0.970	99.79
KNN		93.70	95.32	0.939	99.50
SVM		97.94	98.23	0.980	99.86
RF		90.12	90.34	0.904	90.02
DT	PCA	72.01	68.04	0.71	70.02
KNN		79.56	75.45	0.76	75.40
SVM		88.12	86.32	0.86	86.32
RF		80.0	79.0	0.79	80.0

**Table 6:** *p*-values for DT, SVM, KNN and RF using the RICA- and PCA-based feature extraction methods.

Classifier	Feature Extraction Method	*p*-Value
DT vs. KNN	RICA	0.001
SVM vs. DT		0.000
DT vs. RF		0.021
KNN vs. DT	PCA	0.024
DT vs. SVM		0.031
RF vs. DT		0.03

## Abbreviations

ANN	Artificial Neural Network
ASCII	American Standard Code for Information Interchange
AUC	Area under the Curve
DT	Decision Tree
FDR	False Discovery Rate
FN	False Negative
FNR	False Negative Rate
FP	False Positive
FPR	False Positive Rate
GPU’s	Graphics Processing Unit
HOG	Histogram of Oriented Gradients
KNN	K-Nearest Neighbor
NPV	Negative Predicted Value
PCA	Principal Component Analysis
PPV	Positive Predicted Value
RF	Random Forest
ROC	Receiver Operating Characteristics
SVM	Support Vector Machine
Sn	Sensitivity
Sp	Specificity
TNR	True Negative Rate
TPR	True Positive Rate
